# The Enigmatic Reissner’s Fiber and the Origin of Chordates

**DOI:** 10.3389/fnana.2021.703835

**Published:** 2021-06-23

**Authors:** Francisco Aboitiz, Juan F. Montiel

**Affiliations:** ^1^Departamento de Psiquiatría, Escuela de Medicina, Pontificia Universidad Católica de Chile, Santiago, Chile; ^2^Centro Interdisciplinario de Neurociencias, Pontificia Universidad Católica de Chile, Santiago, Chile; ^3^Centro de Investigación Biomédica, Facultad de Medicina, Universidad Diego Portales, Santiago, Chile

**Keywords:** Reissner’s fiber, chordates, vertebrates, notochord, neural tube, swimming behavior, cerebrospinal fluid

## Abstract

Reissner’s fiber (RF) is a secreted filament that floats in the neural canal of chordates. Since its discovery in 1860, there has been no agreement on its primary function, and its strong conservation across chordate species has remained a mystery for comparative neuroanatomists. Several findings, including the chemical composition and the phylogenetic history of RF, clinical observations associating RF with the development of the neural canal, and more recent studies suggesting that RF is needed to develop a straight vertebral column, may shed light on the functions of this structure across chordates. In this article, we will briefly review the evidence mentioned above to suggest a role of RF in the origin of fundamental innovations of the chordate body plan, especially the elongation of the neural tube and maintenance of the body axis. We will also mention the relevance of RF for medical conditions like hydrocephalus, scoliosis of the vertebral spine and possibly regeneration of the spinal cord.

## Introduction

**FIGURE 1:**
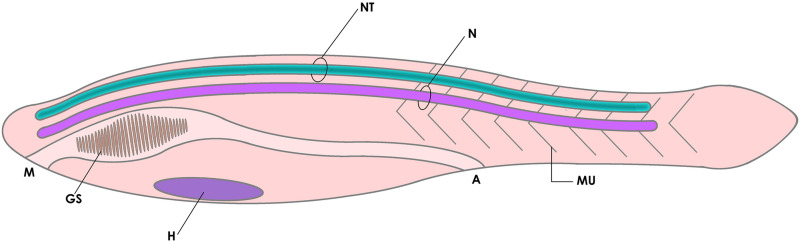
Diagram of the basic elements of the chordate body plan, featuring a tadpole-like animal with a fibrous notochord (N) and a hollow neural tube (NT) in the dorsal side (cavitation is shown in dark green), a muscular tail (MU) for swimming, and gill slits (GS, a character shared with other deuterostomes). A, anus; H, heart; M, mouth.

The phylum Chordata, including lancelets (cephalochordates -Amphioxus), sea squirts (urochordates), and vertebrates, is partly characterized by the presence of a segmented musculature flanking a fibrous notochord that defines a semi-rigid anteroposterior axis, and a muscular post-anal tail used for swimming. Another typical feature of chordates is that the central nervous system is shaped as a hollow neural tube that runs from head to tail, filled with cerebrospinal fluid (CSF) that circulates along the neural canal (NCa) (Figure 1; Wicht and Lacalli, 2005; Aboitiz and Montiel, 2007; Glover and Fritzsch, 2009; Striedter and Northcutt, 2020). The anatomical and behavioral innovations acquired by this group provided the blueprint from which vertebrates emerged and colonized the earth. Nonetheless, the evolutionary origin of chordates themselves remains as one of the great unsolved questions of evolutionary biology (Satoh, 2008; Holland et al., 2015). In this context, the notochord is considered by many a cardinal feature of chordates, being involved in the development of some of the principal characters of this phylum, especially the tailbud and the neural tube (Henrique et al., 2015; Sasai et al., 2021).

In this article, we propose the additional participation of Reissner’s fiber (RF), another highly conserved but much less conspicuous feature of chordates, which probably collaborated with the notochord in the origin of this animal group. RF is a structure secreted to the NCa that arises in early development of most chordates. Despite its phylogenetic preservation and early embryonic origins, the functions of RF have been a matter of discussion for more than a century. Many authors have suggested a role of this fiber in the chemical regulation of the CSF and in the maintenance of the NCa, which together with its phylogenetic conservatism may hint to a participation in the early evolution of the chordate neural tube. Furthermore, recent studies indicate a role of this structure as a proprioceptive organ, providing the necessary muscle tonicity to support a straight body axis in the embryo. In our view, this and other evidence can provide insights on the role of RF in the evolution of chordate’s tail-powered swimming behavior, perhaps the key behavioral characteristic of this phylum. Here we will provide an overview of RF, its comparative structure and its diverse functions, and we will address comparative, clinical, and some recent experimental findings to suggest that this structure was associated with the definition of the body axis and swimming behavior in the early chordates.

## Characterization of Reissner’s Fiber (RF)

As said, RF is an insoluble proteinaceous filament that floats in the CSF inside the chordate NCa. It is secreted by specialized ependymal cells in the mesencephalic-diencephalic (M-D) junction and extends toward the caudal end of the neural tube, where it decomposes and its material enters the meningeal CSF and the bloodstream (Rodríguez et al., 1992, 1998). Since it was first described by Reissner (1860) in the lamprey, it has remained an enigmatic component of the chordate neural tube, to which many functions have been ascribed.

### Composition and Comparative Structure

Reissner’s fiber is present in the NCa of most chordates, including cephalochordates, urochordates, and vertebrates excepting postnatal humans and a few other mammals. It consists of an agglomerate of elastic filaments, mainly composed of the glycoprotein SCO-spondin that contains high amounts of sialic acid, which enhances its adhesive properties (Olsson and Wingstrand, 1954; Olsson, 1972; Holmberg and Olsson, 1984; Rodríguez et al., 1992; Olsson et al., 1994; Gobron et al., 1999). SCO-spondin is a large extracellular matrix molecule containing a multidomain arrangement that includes von Willebrand factor D domains, SCO-spondin repeats, thrombospondin (TSR) domains, and LDL receptor repeats, all components associated with cell adhesion and axonal guidance (Gobron et al., 1999; Meiniel and Meiniel, 2007). There is strong molecular homology of the SCO-spondin gene across species, suggesting that this gene is ancestral to all chordates (Gobron et al., 1999, 2000; Meiniel et al., 2008). Nonetheless, SCO-spondin has lengthened significantly in vertebrates by the addition of repeated TSR domains, which is probably related to its increasingly complex role in promoting axonal growth and neuronal cell differentiation, among other functions (Meiniel et al., 2008). In fact, this protein’s multidomain structure was probably created by domain-shuffling of different gene domains, possibly before the origin of chordates (Kawashima et al., 2009).

**FIGURE 2:**
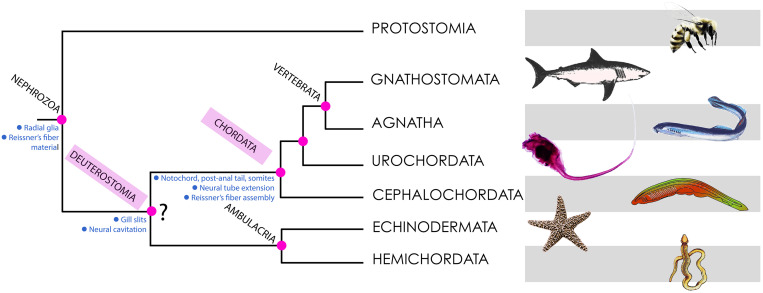
Phylogeny of deuterostomes, indicating the points of origin of the characters discussed in this article. Most phylogenetic analyses place Deuterostomia as a monophyletic group (as depicted here), with Chordata and ambulacria as sister groups. However, some recent findings using large scale genetic databases claim that there is no evidence for Deuterostomia as a monophyletic group (Kapli et al., 2021). Hence the interrogation mark shown at this node. The main conclusions of this article are consistent with both views.

Some findings point to the existence of genes orthologous to SCO-spondin in echinoderms and hemichordates (both representing the sister group of chordates according to some authors; Figure 2) and possibly in all bilaterian animals, suggesting that RF-like material (but not a polymerized RF) was secreted by radial glia-like cells in the common ancestor of Bilateria (Gobron et al., 1999; Meiniel et al., 2008; Mashanov et al., 2009; Helm et al., 2017). Arendt et al. (2015) proposed that RF material derives from the secretion of mucociliary cells of ancestral metazoans (exemplified by present-day placozoans) that move food particles into the extracellular digestive cavity (Arendt et al., 2015).

### Sites of Production

**FIGURE 3:**
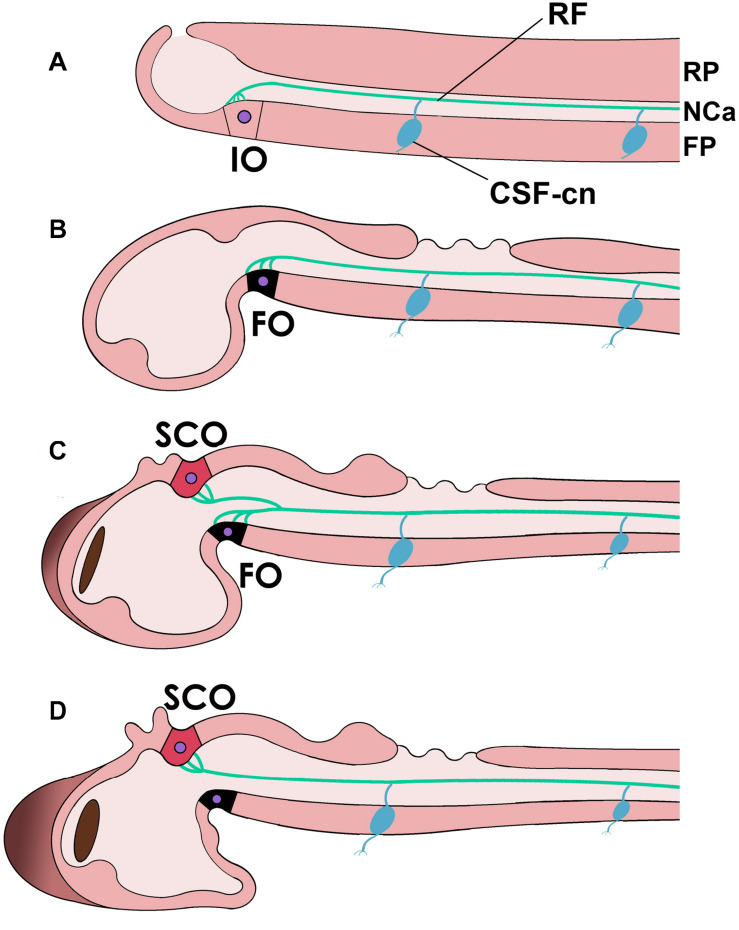
Reissner’s fiber (RF, green) in different chordates and during development. **(A)** In the neural tube of cephalochordates RF is secreted by the infundibular organ of the floor plate (FP) (IO) and floats in the CSF-filled neural or central canal (NCa). **(B–D)** Reissner’s fiber in teleost fish [**(B)**, early embryonic; **(C)**, middle stage; **(D)**, advanced]. Note that in teleosts, the flexural organ (FO, possibly homologous to the IO in cephalochordates) is replaced by the subcommissural organ (SCO) in the roof plate (RP) as the secreting organ of RF. Note also the CSF-contacting neurons (CSF-cn) with an axon (inferior process) and a dendrite or cilium that enters the CSF and contacts RF.

In late vertebrate development, RF is secreted by the subcommissural organ (SCO), a dorsal circumventricular organ located below the posterior commissure in the dorsal neural tube (the roof plate) at the M-D junction (dorsal prosomer P1; Nieuwenhuys, 1988; Meiniel et al., 1996; Grondona et al., 2012; Puelles, 2018; Muñoz et al., 2019; Diaz and Puelles, 2020; Figure 3). The SCO has a complex histological structure containing specialized, elongated ependymal cells with a basal process contacting the perivascular space that receives synaptic connections and an apical process that reaches the central canal and secretes RF, among other components (Rodríguez et al., 1992). SCO secretion includes RF and other components like the thyroid hormone transporter transthyretrin, basic fibroblast growth factor and other glycoproteins, some of which are soluble and reach the bloodstream via the ependymal cell’s basal end, or the CSF via the cell’s apical extreme. RF formation in the SCO’s apical surface involves the “packaging” of the secreted proteins by disulfide bonds to form an insoluble thread that grows along the NCa (Rodríguez et al., 1992; Vio et al., 2008; Kiecker, 2018). However, SCO is not the only site of RF production during embryogenesis. In the early embryos of several vertebrates from fish to mammals, RF material is first secreted by the (ventral) floor plate cells, together with similar proteins like F-spondin. Later, RF material becomes secreted by a specialized flexural organ (FO) of the ventral M-D cephalic flexure (prosomers P1 and/or P2) and assembled into RF. Only in later stages, the SCO starts contributing to RF, together with the FO until the latter ceases its function, and only the SCO produces RF in the adult (Oksche, 1969; Lichtenfeld et al., 1999; Meiniel et al., 2008).

This developmental sequence seems to match the phylogenetic history of RF production. In cephalochordates, RF is secreted by the infundibular organ (IO), located in the diencephalic floor plate (Olsson and Wingstrand, 1954; Olsson, 1972; Olsson et al., 1994; Wicht and Lacalli, 2005; Figure 3). Based on possessing highly similar secretory mechanisms and topographic position (both are located in the site corresponding to the cephalic flexure, likely in prosomer P1/P2), the embryonic FO has been considered to be homologous to the cephalochordate IO (Olsson and Wingstrand, 1954; Oksche, 1969; Olsson, 1972; Rodríguez et al., 1992; Olsson et al., 1994; Wicht and Lacalli, 2005). That is, both structures may derive from a structure present in the last common ancestor of vertebrates and cephalochordates.

Notably, larval urochordates also produce a RF-like structure that in some species is secreted by a specialized fibrinogen cell (FC) in the tail neural cord, which has been compared to the cephalochordate IO by virtue of secreting the same material (Holmberg and Olsson, 1984). Phylogenetic analyses indicate that urochordates are the sister group of vertebrates, while cephalochordates represent this phylum’s earliest branch (Bourlat et al., 2006). If this view is correct, the Amphioxus’ IO may be closer to the ancestral RF secreting organ than the urochordate FC, the latter being a derived structure associated with the sessile lifestyle of urochordates as adults.

All three organs (IO, FC, and SCO) are located at the M-D junction in the different species (as said, prosomer P1), either in the floor plate (IO, FC) or in the roof plate (SCO). In recent years, several genes have been found to control SCO development and RF production (as well as the development of other organs too), including Pax6, SOX3, L1-CAM, and apoptosis-related genes (Estivill-Torrús et al., 2001; Ramos et al., 2004; Carmona-Calero et al., 2009; Lee et al., 2012; Matsumoto et al., 2020; Yang et al., 2021). It is possible that the expression of genes involved in IO (or FO) differentiation in the floor plate of the M-D junction became co-opted in the roof plate during the origin of vertebrates, for reasons yet unknown. If this is correct, these structures, IO/FO on the one hand and SCO on the other, might qualify as serial homologs of each other (serial homology is a term used for repeated body structures like limbs or hairs; Montiel and Aboitiz, 2018). In other words, in the origin of vertebrates the ancestral developmental program involved in ventral RF secretion became activated in a different region, in the dorsal aspect of the M-D junction. As mentioned, the reasons for this transition remain as another mystery.

## Hypotheses About RF’s Functions

Despite all the accumulated knowledge about RF, there is no clarity about its primary, fundamental function, or adaptive significance (Rodríguez et al., 1992; Meiniel et al., 2008; Bearce and Grimes, 2021). In association with ciliary beating from ependymal cells, RF has been related to the circulation and ionic homeostasis of the CSF, where its high sialic acid contents enable it to bind CSF substances and transport them along the neural canal as the fiber grows. Mainly, RF may be involved in the transport and regulation of CSF monoamines, especially adrenaline, which regulates SCO secretion. Likewise, the SCO has been proposed to participate in osmoregulation, sodium excretion, diuresis, and water intake, although these findings remain controversial (Rodríguez et al., 1992; Muñoz et al., 2019).

### Early Embryogenesis: Maintenance of the Neural Canal

Hydrocephalus is a clinical condition where CSF accumulates in the cerebral ventricles due to neural tube defects that impair CSF circulation. Dysfunction of the SCO-RF complex has been associated with hydrocephalus in several species, including humans. Immunological damage to the SCO or absence of RF result in the collapse of the cerebral aqueduct (the NCa at the M-D junction) which blocks CSF flow, consequently increasing intraventricular pressure in the cerebral hemispheres (Overholser et al., 1954; Vio et al., 2000; Pérez-Fígares et al., 2001). The postnatal hydrocephalus hyh mouse develops absence of the NCa in the spinal cord and stenosis of the anterior mesencephalic aqueduct, as well as an impaired SCO and absence of RF (Irigoin et al., 1990). In this mutant, it was found that the ependyma’s embryonic denudation starts in the ventral side and progresses dorsally during development, before the onset of postnatal hydrocephalus. The loss of floor plate cells and the lack of RF in this mutant may produce a distortion of the ependyma that collapses due to pressure produced by the brain parenchyma (Jiménez et al., 2001; Wagner et al., 2003).

### Roles in Later Neuronal Development and Function

The SCO-RF complex not only has a role in early development but also interacts with the nervous system in diverse ways. During vertebrate development, the SCO and its secreted proteins have been associated with axonal guidance in the neural tube midline, a character shared by the roof and floor plates in the embryonic neural tube (Meiniel et al., 1996; Gobron et al., 2000; Grondona et al., 2012). RF has been also related to regeneration of the caudal neural tube in lower vertebrates (Meiniel et al., 1996; Alibardi, 2021), and soluble RF material secreted by the SCO has been proposed to contribute to adult neurogenesis in mammals (Guerra et al., 2015). In addition, the SCO is a highly innervated organ, receiving different inputs carrying monoamines, acetylcholine, neuropeptides, and GABA. Some findings suggest that serotonin and other monoamines may modulate SCO secretory activity, perhaps driven by signals generated from the neural tube itself (Rodríguez et al., 1992; Richter et al., 2004; El Hiba et al., 2020).

### CSF-Contacting Neurons

Perhaps of more relevance to this article, the NCa walls contain abundant CSF-contacting neurons (CSF-cn) all along its length (Rodríguez et al., 1992; Bearce and Grimes, 2021). CSF-cn project dendrite-like extensions into the NCa and extend a large cilium and several stereocilia that contact RF. Although some of these neurons are apparently GABAergic, they are also positive for several other neurotransmitters, including monoamines, somatostatin (SST), and Urotensin II-related peptide (URP). URP neurons are innervated by SST+ axons, while SST+ neurons receive serotoninergic innervation (Rodríguez et al., 1992; Bearce and Grimes, 2021). These neurons extend axons to the lateroventral spinal cord and other regions and have been proposed to provide a feedback mechanism where CSF-cn may control the SCO activity (Rodríguez et al., 1992). CSF-cn, therefore, are in a position to sense a variety of chemical and mechanical stimuli from the CSF and RF, including bending of the RF and body axis and stimulating the dorsal musculature to correct these deviations (Böhm et al., 2016; Jalalvand et al., 2016). The latter suggests a role in locomotion and body position of CSF-cn and RF (Gobron et al., 1999, 2000), an issue that will be discussed below.

### Development of a Straight Body Axis

Additionally, several studies suggest that RF alterations are involved in the pathogenesis of scoliosis. In this clinical condition, the vertebral spine curves sideways instead of maintaining a straight axis, affecting posture and worsening with age. Early in the last century, Nicholls (1913) and Kolmer (1921) proposed that RF could work as an axial proprioceptive organ, providing somatosensory control over the body and contributing to maintaining the body axis in the larval stage. Other studies performed last century had shown that SCO or RF disruption produced a distortion of the body axis in larval amphibians and fish (see Rodríguez et al., 1992). Additional evidence indicated that disruption of ependymal ciliary movements impairs CSF flow and produce curvature of the spine in zebrafish, suggesting a relation between ciliary movement and RF function (Brand et al., 1996; Kramer-Zucker et al., 2005; Bearce and Grimes, 2021). More recent studies performed in the zebrafish supported this possibility, demonstrating that RF is critical for maintaining a straight body axis and vertebral spine morphogenesis, as the absence of RF during development leads to scoliosis in the adult (Driever, 2018; Ringers and Jurisch-Yaksi, 2020). In a first study, Cantaut-Belarif et al. (2018) eliminated RF in mutants lacking SCO-spondin. On the first day after hatching, mutant larvae had a normally straight body axis (perhaps maintained by the nascent notochord), but after 30 h, larvae displayed a trunk’s distorted curvature. As mentioned, a similar phenotype has been observed in animals with ciliary movement defects in the ependymal cells lining the neural canal, which impairs CSF circulation (Bearce and Grimes, 2021). However, ciliogenesis and CSF circulation are normal in SCO-spondin mutants. On the other hand, in mutants with ciliary defects, RF does not form. Cantaut-Belarif et al. (2018) concluded that ciliary movement is needed to form RF and that RF, but not ciliary movement, is needed to maintain the body axis straight through larval development. In a subsequent study, hypomorphic SCO-spondin mutant larvae (expressing lower protein levels and forming a defective RF) produced a curved tail and scoliosis in the adult (Troutwine et al., 2020).

Another study demonstrated that RF stimulates mechanosensory cilia of CSF-cn in the spinal canal. When the axis is straight, RF floats in the neural canal, but when it curves comes into contact with mechanosensory neurons in the neural tube’s inner wall that activate axial musculature, restoring the body’s stiffness axis (Orts-Del’Immagine et al., 2020). Two further articles have shed additional insight into RF- CSF-cn mechanosensory transduction (Cantaut-Belarif et al., 2020; Lu et al., 2020). These studies showed that URP requires an intact RF to be expressed and is downregulated in SCO-spondin mutants, which results in impaired signaling to trunk muscles. Furthermore, treating mutants with epinephrine increased URP expression and rescued the phenotype, suggesting that this neurotransmitter mediates RF-CSF-cn signaling, perhaps via calcium transients in the spinal neurons. Furthermore, providing URP to the CSF resulted in a restoration of the larval and adult mutants’ phenotype. Finally, SCO-spondin mutants also develop increased scoliosis-associated neuroinflammatory responses. Suppressing inflammation results in restoration of the wild-type phenotype (Rose et al., 2020). In discussing these findings, Ringers and Jurisch-Yaksi (2020) hypothesized that RF protects the spinal cord from inflammation and impairs normal maintenance of a straight body axis by inhibiting mechanosensory reflexes that maintain a stiff body posture.

## Discussion: Role of RF in Early Chordate Evolution

**FIGURE 4:**
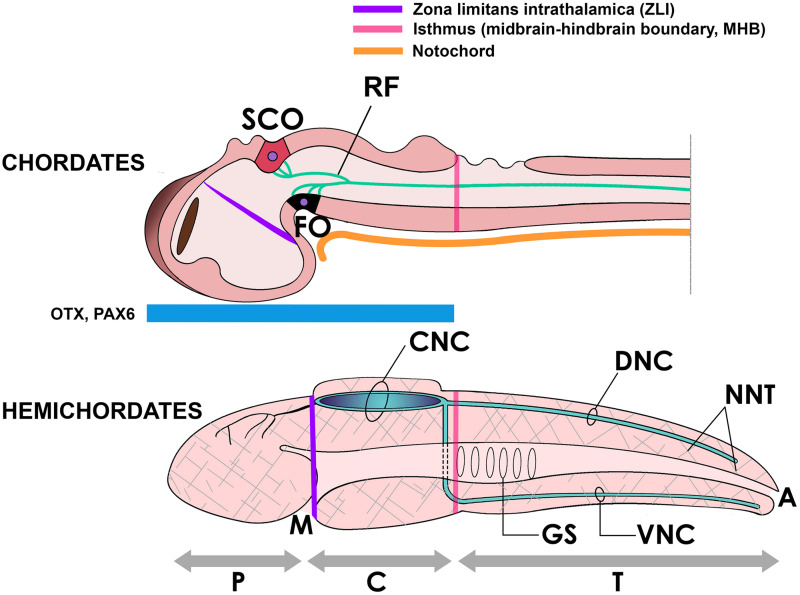
Above, the neural tube of a chordate (embryonic fish) showing the subcommissural organ (SCO) and the flexural organ (FO), sites of origin of RF. These organs are located in the mesencephalic-diencephalic boundary, which is included in the embryonic region bound by the zona limitans intrathalamica (ZLI) anteriorly and the midbrain-hindbrain boundary (MHB) posteriorly. This region is strongly Otx-positive in early development and for this reason has been considered homologous to the location of the collar region (C) of the hemichordate body (below), displaying the same morphogenetic boundaries (ZLI and MHB homologs) and containing a hollow collar nerve cord (CNC) (Pani et al., 2012; Lowe et al., 2015). Beside the CNC, hemichordates display dorsal and ventral nerve cords (DNC, VNC) in the body trunk (T) and a diffuse neural network (NNT, hatched areas) covering the body (Holland et al., 2015). A, anus; GS, gill slits; M, mouth; P, proboscis.

As we have discussed above, despite much research being performed on RF there is no clarity yet about its primary role in neural development or in chordate evolution. Since RF and the SCO are required to prevent the collapse of the cerebral aqueduct of vertebrates, and a polymerized RF exists only in chordates, some authors have asked whether these components (more precisely, RF and the cephalochordate IO) might have contributed to the formation of the neural tube in chordate origins (Meiniel et al., 2008). However, a hollow nervous system may not be a chordate-specific character. The nerve cord of hemichordates (Figures 2, 4) displays a localized cavitation that for many authors foreshadows the chordate neural tube (Kaul and Stach, 2010), although there are other opinions (Satoh, 2008). In any case, the origin of neurulation remains an unsolved problem. An early proposal prescribed that neurulation resulted from the invagination of two longitudinal ciliary bands present in the hypothetical ancestral dipleurula larva (Garstang, 1894; Holland, 2011). While being an appealing hypothesis, there is no evidence in its support yet. It is also possible that the secretion of non-polymerized SCO-spondin proteins in the apical neuroectoderm contributed to the early invagination of the nerve cord in early deuterostomes or chordates (Meiniel et al., 2008), although again more evidence in this line is still needed.

Of note, the hemichordate neural tube is located in the collar region of the head, a region strongly positive for the genes Otx and Pax6, whose counterpart in chordates includes the M-D junction, according to gene expression mapping studies (Figure 4; Williams and Holland, 1996; Gobron et al., 1999; Holland and Short, 2008; Pani et al., 2012; Lowe et al., 2015). One possibility is that in chordate origins, the assembly of RF was associated with the development of an extended NCa running not only in anterior regions but also in caudal Otx-negative regions of the ancestral nerve cord; that is, into the body trunk and the post-anal tail. Embryonic elongation of the neural tube is a fundamental process shaping the chordate body plan (Lacalli, 2000). Yet, in lower vertebrates including ray finned fish and the lamprey, neural tube cavitation does not seem to occur at once but there is first a solid medullary cord (the neural keel) that cavitates at different levels along its length, becoming a continuous neural canal only later in development (Handrigan, 2003). Recalling the role of RF in preventing hydrocephalus, this structure may have participated in the production and maintenance of a continuous NCa along the longitudinal body axis in these species. In addition, it is of interest to recall that the posterior spinal cord innervating the chordate tail is produced by secondary neurulation, i.e., the formation of a secondary neural tube derived from tail bud mesenchyme, that later coalesces with the main neural tube (Handrigan, 2003; Beaster-Jones et al., 2008; Henrique et al., 2015). In cephalochordates, urochordates and vertebrates the tail’s NCa contains a RF (Obermüller-Wilén and Olsson, 1974), and a RF is also present in the regenerating tail of lepidosaurians and other vertebrates (Meiniel et al., 1996; Alibardi, 2021). Therefore, it would be highly interesting to investigate whether RF contributed to the elongation and maintenance of the secondary neural canal in the tailbud of early chordates.

Beside its possible role in early chordate embryogenesis, RF may have been also crucial for the development of chordate swimming behavior, which eventually turned out to be the key for the success of vertebrates. In view of the recent evidence indicating a role of RF in the development of a straight axis, we tentatively suggest that in early chordates, the presence of a mechanically passive notochord might not have been sufficient to maintain the rigidness of the anteroposterior axis as the embryo grew in size. Stimulation of the notochord’s contractile elements and of the dorsal musculature was required to maintain a straight body, which was provided by the RF/CSF-cn proprioceptive circuit. In other words, RF in contact with CSF-cn (expressing URP or other neurotransmitters), may have provided a body position sensor that maintained the necessary tightness of the notochord and dorsal musculature in the growing larva, maximizing elastic energy during swimming strokes. Summarizing, elongation of the NCa into the trunk and tail regions, containing inside RF in contact with CSF-cn, may have been a key acquisition of early chordates that enabled not only the maintenance of a straight axis but may have also contributed to the neural control required for efficient swimming.

Notably, recent studies have shed light on another highly conserved event in early chordate development: a slight ventral bending of the embryonic tail during early development to fit the embryonic membranes’ curvature. While this had been considered to result from external forces imposed to the growing embryo, it was found that actomyosin accumulates in the ventral side of the notochord, a condition that is required for proper bending of the tail (Lu et al., 2020). Whether RF and the CSF-cn participate in this process is also a matter for further research.

## Final Comments

This article has reviewed some old and recent findings regarding RF function from an evolutionary perspective. We hypothesize that the assembly of RF as a growing filament in the rudimentary NCa of chordate ancestors contributed to the extension of the neural tube into the tail region in concomitance with the differentiation of the notochord. This innovation also provided proprioceptive control to the body trunk and tail, facilitating the maintenance of a straight axis and the development of a contractile swimming tail. RF was first discovered some 160 years ago, and its function and strict conservatism across all chordates have remained an evolutionary mystery. The recent findings may provide some critical insight into its primary function during early chordate development and its maintenance in the phylum. Further research aimed at unveiling a possible participation of RF or its unpolymerized components in the process of neurulation, as well as in the role of this structure in the development of the body axis and swimming behavior may shed some further light into the origins of this intriguing neural tube component. Finally, understanding the role of RF in neural development may contribute to the treatment of spinal cord disorders, including hydrocephalus and scoliosis. In addition, considering a possible participation of RF in the lengthening of the neural canal and tail regeneration in lower vertebrates, further research could be directed to unveil the therapeutic involvement of this structure in spinal cord regeneration after injury or degenerative disease.

## Author Contributions

All authors listed have made a substantial, direct and intellectual contribution to the work, and approved it for publication.

## Conflict of Interest

The authors declare that the research was conducted in the absence of any commercial or financial relationships that could be construed as a potential conflict of interest.

## References

[B1] Aboitiz F. Montiel J. (2007). Origin and evolution of the vertebrate telencephalon, with special reference to the mammalian neocortex. *Adv. Anat. Embryol. Cell Biol*. 193 1–112. 10.1007/978-3-540-49761-5 17595827

[B2] Alibardi L. (2021). Tail regeneration in Lepidosauria as an exception to the generalized lack of organ regeneration in amniotes. *Exp. Zool. B. Mol. Dev. Evol.* 336 145–164. 10.1002/jez.b.22901 31532061

[B3] Arendt D. Benito-Gutierrez E. Brunet T. Marlow H. (2015). Gastric pouches and the mucociliary sole: setting the stage for nervous system evolution. *Philos. Trans. R. Soc. Lond. B Biol. Sci.* 370:20150286. 10.1098/rstb.2015.0286 26554050 PMC4650134

[B4] Bearce E. A. Grimes D. T. (2021). On being the right shape: roles for motile cilia and cerebrospinal fluid flow in body and spine morphology. *Semin. Cell Dev. Biol*. 110 104–112. 10.1016/j.semcdb.2020.07.005 32693941

[B5] Beaster-Jones L. Kaltenbach S. L. Koop D. Yuan S. Chastain R. Holland L. Z. (2008). Expression of somite segmentation genes in amphioxus: a clock without a wavefront? *Dev. Genes Evol.* 218 599–611. 10.1007/s00427-008-0257-5 18949486

[B6] Böhm U. L. Prendergast A. Djenoune L. Nunes Figueiredo S. Gomez J. Stokes C. (2016). CSF-contacting neurons regulate locomotion by relaying mechanical stimuli to spinal circuits. *Nat. Commun.* 7:10866. 10.1038/ncomms10866 26946992 PMC4786674

[B7] Bourlat S. J. Juliusdottir T. Lowe C. J. Freeman R. Aronowicz J. Kirschner M. (2006). Deuterostome phylogeny reveals monophyletic chordates and the new phylum Xenoturbellida. *Nature* 444 85–88. 10.1038/nature05241 17051155

[B8] Brand M. Heisenberg C. P. Warga R. M. Pelegri F. Karlstrom R. O. Beuchle D. (1996). Mutations affecting development of the midline and general body shape during zebrafish embryogenesis. *Development* 123 129–142. 9007235 10.1242/dev.123.1.129

[B9] Cantaut-Belarif Y. Orts Del’Immagine A. Penru M. Pézeron G. Wyart C. Bardet P. L. (2020). Adrenergic activation modulates the signal from the Reissner fiber to cerebrospinal fluid-contacting neurons during development. *Elife* 9:e59469. 10.7554/eLife.59469 33048048 PMC7591253

[B10] Cantaut-Belarif Y. Sternberg J. R. Thouvenin O. Wyart C. Bardet P. L. (2018). The reissner fiber in the cerebrospinal fluid controls morphogenesis of the body axis. *Curr. Biol.* 28 2479.e4–2486.e4. 30057305 10.1016/j.cub.2018.05.079 PMC6089837

[B11] Carmona-Calero E. M. González-Marrero I. González-Toledo J. M. Castañeyra-Ruiz A. De Paz-Carmona H. Castañeyra-Ruiz L. (2009). Reissner’s fibre proteins and p73 variations in the cerebrospinal fluid and subcommissural organ of hydrocephalic rat. *Anat. Histol. Embryol*. 38 282–285. 10.1111/j.1439-0264.2009.00939.x 19519738

[B12] Diaz C. Puelles L. (2020). Developmental genes and malformations in the hypothalamus. *Front. Neuroanat.* 14:607111. 10.3389/fnana.2020.607111 33324176 PMC7726113

[B13] Driever W. (2018). Developmental biology: reissner’s fiber and straightening of the body axis. *Curr. Biol*. 28 R833–R835. 10.1016/j.cub.2018.05.080 30086316

[B14] El Hiba O. Draoui A. Gamrani H. (2020). The neuroactive neurosteroid Dehydroepiandrosterone Sulfate (DHEAS) modulates the serotonergic system within the dorsal Raphe nucleus and the cerebrospinal fluid release of Reissner’s fiber in rat. *C. R. Biol*. 343 101–110. 10.5802/crbiol.3 32720492

[B15] Estivill-Torrús G. Vitalis T. Fernández-Llebrez P. Price D. J. (2001). The transcription factor Pax6 is required for development of the diencephalic dorsal midline secretory radial glia that form the subcommissural organ. *Mech. Dev*. 109 215–224. 10.1016/s0925-4773(01)00527-5 11731235

[B16] Garstang W. (1894). Preliminary note on a new theory of the phylogeny of the Chordata. *Zool. Anz*. 17 122–125.

[B17] Glover J. C. Fritzsch B. (2009). “Brains of primitive chordates,” in *Encyclopedia of Neuroscience*, 4th Edn, Vol. 6 ed. Squire L. (Oxford: Academic Press), 439–448.

[B18] Gobron S. Creveaux I. Meiniel R. Didier R. Dastugue B. Meiniel A. (1999). SCO-spondin is evolutionarily conserved in the central nervous system of the chordate phylum. *Neuroscience* 88 655–664. 10.1016/s0306-4522(98)00252-8 10197783

[B19] Gobron S. Creveaux I. Meiniel R. Didier R. Herbet A. Bamdad M. (2000). Subcommissural organ/Reissner’s fiber complex: characterization of SCO-spondin, a glycoprotein with potent activity on neurite outgrowth. *Glia* 32 177–191. 10.1002/1098-1136(200011)32:2<177::aid-glia70<3.0.co;2-v 11008217

[B20] Grondona J. M. Hoyo-Becerra C. Visser R. Fernández-Llebrez P. López-Ávalos M. D. (2012). The subcommissural organ and the development of the posterior commissure. *Int. Rev. Cell Mol. Biol*. 296 63–137. 10.1016/B978-0-12-394307-1.00002-3 22559938

[B21] Guerra M. M. González C. Caprile T. Jara M. Vío K. Muñoz R. I. (2015). Understanding how the subcommissural organ and other periventricular secretory structures contribute via the cerebrospinal fluid to neurogenesis. *Front. Cell Neurosci*. 9:480. 10.3389/fncel.2015.00480 26778959 PMC4689152

[B22] Handrigan G. R. (2003). Concordia discors: duality in the origin of the vertebrate tail. *J. Anat.* 202 255–267. 10.1046/j.1469-7580.2003.00163.x 12713266 PMC1571085

[B23] Helm C. Karl A. Beckers P. Kaul-Strehlow S. Ulbricht E. Kourtesis I. (2017). Early evolution of radial glial cells in Bilateria. *Proc. Biol. Sci.* 284:20170743. 10.1098/rspb.2017.0743 28724733 PMC5543218

[B24] Henrique D. Abranches E. Verrier L. Storey K. G. (2015). Neuromesodermal progenitors and the making of the spinal cord. *Development* 142 2864–2875. 10.1242/dev.119768 26329597 PMC4958456

[B25] Holland L. Z. Short S. (2008). Gene duplication, co-option and recruitment during the origin of the vertebrate brain from the invertebrate chordate brain. *Brain Behav. Evol*. 72 91–105. 10.1159/000151470 18836256

[B26] Holland N. D. (2011). Walter Garstang: a retrospective. *Theory Biosci*. 130 247–258. 10.1007/s12064-011-0130-3 21833594

[B27] Holland N. D. Holland L. Z. Holland P. W. (2015). Scenarios for the making of vertebrates. *Nature* 520 450–455. 25903626 10.1038/nature14433

[B28] Holmberg K. Olsson R. (1984). The origin of Reissner’s fibre in an appendicularian, Oikopleura dioica. *Vidensk. Meddr Dansk Naturh. Foren*. 145 43–52.

[B29] Irigoin C. Rodríguez E. M. Heinrichs M. Frese K. Herzog S. Oksche A. (1990). Immunocytochemical study of the subcommissural organ of rats with induced postnatal hydrocephalus. *Exp. Brain Res*. 82 384–392. 10.1007/BF00231257 1704848

[B30] Jalalvand E. Robertson B. Wallén P. Grillner S. (2016). Ciliated neurons lining the central canal sense both fluid movement and pH through ASIC3. *Nat. Commun*. 7:10002. 10.1038/ncomms10002 26743691 PMC4729841

[B31] Jiménez A. J. Tomé M. Páez P. Wagner C. Rodríguez S. Fernández-Llebrez P. (2001). A programmed ependymal denudation precedes congenital hydrocephalus in the hyh mutant mouse. *J. Neuropathol. Exp. Neurol*. 60 1105–1119. 10.1093/jnen/60.11.1105 11706940

[B32] Kapli P. Natsidis P. Leite D. J. Fursman M. Jeffrie N. Rahman I. A. (2021). Lack of support for Deuterostomia prompts reinterpretation of the first Bilateria. *Sci. Adv*. 7:eabe2741. 10.1126/sciadv.abe2741 33741592 PMC7978419

[B33] Kaul S. Stach T. (2010). Ontogeny of the collar cord: neurulation in the hemichordate Saccoglossus kowalevskii. *J. Morphol*. 271 1240–1259. 10.1002/jmor.10868 20665533

[B34] Kawashima T. Kawashima S. Tanaka C. Murai M. Yoneda M. Putnam N. H. (2009). Domain shuffling and the evolution of vertebrates. *Genome Res*. 19 1393–1403. 10.1101/gr.087072.108 19443856 PMC2720177

[B35] Kiecker C. (2018). The origins of the Circumventricular organs. *J. Anat*. 232 540–553. 10.1111/joa.12771 29280147 PMC5835788

[B36] Kolmer W. (1921). Das Sagittalorgan der wirbeltiere. *Zeitschrift Anat. Entwicklungsgesch*. 60 652–717.

[B37] Kramer-Zucker A. G. Olale F. Haycraft C. J. Yoder B. K. Schier A. F. Drummond I. A. (2005). Cilia-driven fluid flow in the zebrafish pronephros, brain and Kupffer’s vesicle is required for normal organogenesis. *Development* 132 1907–1921. 10.1242/dev.01772 15790966

[B38] Lacalli T. C. (2000). Cell morphology in amphioxus nerve cord may reflect the time course of cell differentiation. *Int. J. Dev. Biol.* 44 903–906. 11206331

[B39] Lee K. Tan J. Morris M. B. Rizzoti K. Hughes J. Cheah P. S. (2012). Congenital hydrocephalus and abnormal subcommissural organ development in Sox3 transgenic mice. *PLoS One* 7:e29041. 10.1371/journal.pone.0029041 22291885 PMC3266892

[B40] Lichtenfeld J. Viehweg J. Schutzenmeister J. Naumann W. W. (1999). Reissner’s substance expressed as a transient pattern in vertebrate floor plate. *Anat. Embryol. (Berl).* 200 161–174. 10.1007/s004290050270 10424874

[B41] Lowe C. J. Clarke D. N. Medeiros D. M. Rokhsar D. S. Gerhart J. (2015). The deuterostome context of chordate origins. *Nature* 520 456–465. 10.1038/nature14434 25903627

[B42] Lu H. Shagirova A. Goggi J. L. Yeo H. L. Roy S. (2020). Reissner fibre-induced urotensin signalling from cerebrospinal fluid-contacting neurons prevents scoliosis of the vertebrate spine. *Biol. Open* 9:bio052027. 10.1242/bio.052027 32409296 PMC7240301

[B43] Mashanov V. S. Zueva O. R. Heinzeller T. Aschauer B. Naumann W. W. Grondona J. M. (2009). The central nervous system of sea cucumbers (Echinodermata: holothuroidea) shows positive immunostaining for a chordate glial secretion. *Front. Zool.* 6:11. 10.1186/1742-9994-6-11 19538733 PMC2705372

[B44] Matsumoto Y. Yamaguchi Y. Hamachi M. Nonomura K. Muramatsu Y. Yoshida H. (2020). Apoptosis is involved in maintaining the character of the midbrain and the diencephalon roof plate after neural tube closure. *Dev. Biol.* 2020 101–109. 10.1016/j.ydbio.2020.09.015 32979334

[B45] Meiniel A. Meiniel R. Didier R. Creveaux I. Gobron S. Monnerie H. (1996). The subcommissural organ and Reissner’s fiber complex. An enigma in the central nervous system? *Prog. Histochem. Cytochem.* 30 1–66. 10.1016/s0079-6336(96)80015-5 8824845

[B46] Meiniel O. Meiniel A. (2007). The complex multidomain organization of SCO-spondin protein is highly conserved in mammals. *Brain Res. Rev.* 53 321–327. 10.1016/j.brainresrev.2006.09.007 17126404

[B47] Meiniel O. Meiniel R. Lalloué F. Didier R. Jauberteau M. O. Meiniel A. (2008). The lengthening of a giant protein: when, how, and why? *J. Mol. Evol.* 66 1–10. 10.1007/s00239-007-9055-3 18046595

[B48] Montiel J. F. Aboitiz F. (2018). Homology in amniote brain evolution: the rise of molecular evidence. *Brain Behav. Evol.* 91 59–64. 10.1159/000489116 29860258

[B49] Muñoz R. I. Kähne T. Herrera H. Rodríguez S. Guerra M. M. Vío K. (2019). The subcommissural organ and the Reissner fiber: old friends revisited. *Cell Tissue Res.* 375 507–529. 10.1007/s00441-018-2917-8 30259139

[B50] Nicholls G. E. (1913). The structure and development of Reissner’s fibre and subcommissural organ. *Q. J. Microsc. Sci.* 58(Part 1) 1–116.

[B51] Nieuwenhuys R. (1988). “Amphioxus,” in *The Central Nervous System of Vertebrates*, eds Nieuwenhuys R. Ten Donkelaar H. J. Nicholson C. (Berlin: Springer), 365–396.

[B52] Obermüller-Wilén H. Olsson R. (1974). The Reissner’s fiber termination in some lower chordates. *Acta Zool.* 55 71–79.

[B53] Oksche A. (1969). The subcommissural organ. *J. Neurovisc. Relat.* 31(Suppl. 9) 111+. 10.1007/978-3-662-25519-3_6 4919437

[B54] Olsson R. (1972). Reissner’s fiber in ascidian tadpole larvae. *Acta Zool. Stockh*. 53 17–21.

[B55] Olsson R. Wingstrand K. G. (1954). Reissner’s fiber and the infundibular organ in Amphioxus. *Univ. Bergen Arbok (Publ. Biol. Stat.)* 14 1–14.

[B56] Olsson R. Yulis R. Rodriguez E. M. (1994). The infundibular organ of the lancelet (Branchiostoma lanceolatum Acrania): an immunocytochemical study. *Cell Tiss. Res*. 277 107–114.

[B57] Orts-Del’Immagine A. Cantaut-Belarif Y. Thouvenin O. Roussel J. Baskaran A. Langui D. (2020). Sensory neurons contacting the cerebrospinal fluid require the Reissner fiber to detect spinal curvature in vivo. *Curr. Biol.* 30 827.e4–839.e4. 10.1016/j.cub.2019.12.071 32084399

[B58] Overholser M. D. Whitley J. R. O’Dell B. L. Hogan A. G. (1954). The ventricular system in hydrocephalic rat brains produced by a deficiency of vitamin B12 or folic acid in the maternal diet. *Anat. Rec.* 120 917–933. 14350261 10.1002/ar.1091200407

[B59] Pani A. M. Mullarkey E. E. Aronowicz J. Assimacopoulos S. Grove E. A. Lowe C. J. (2012). Ancient deuterostome origins of vertebrate brain signalling centres. *Nature* 483 289–294. 10.1038/nature10838 22422262 PMC3719855

[B60] Pérez-Fígares J. M. Jimenez A. J. Rodríguez E. M. (2001). Subcommissural organ, cerebrospinal fluid circulation, and hydrocephalus. *Microsc. Res. Tech.* 52 591–607. 10.1002/1097-0029(20010301)52:5<591::aid-jemt1043<3.0.co;2-7 11241868

[B61] Puelles L. (2018). Developmental studies of avian brain organization. *Int. J. Dev. Biol.* 62 207–224. 10.1387/ijdb.170279LP 29616730

[B62] Ramos C. Fernández-Llebrez P. Bach A. Robert B. Soriano E. (2004). Msx1 disruption leads to diencephalon defects and hydrocephalus. *Dev. Dyn.* 230 446–460. 10.1002/dvdy.20070 15188430

[B63] Reissner E. (1860). Beiträge zur Kenntnis vom Bau des Rückenmarkes von Petromyzon fluviatilis L. *Arch. Anat. Physiol. Wiss Med. (Leipzig).* 77 545–588.

[B64] Richter H. G. Tomé M. M. Yulis C. R. Vío K. J. Jiménez A. J. Pérez-Fígares J. M. (2004). Transcription of SCO-spondin in the subcommissural organ: evidence for down-regulation mediated by serotonin. *Brain Res. Mol. Brain Res.* 129 151–162. 10.1016/j.molbrainres.2004.07.003 15469891

[B65] Ringers C. Jurisch-Yaksi N. (2020). Development: how the reissner fiber keeps our back straight. *Curr. Biol.* 30 R705–R708. 10.1016/j.cub.2020.04.073 32574632

[B66] Rodríguez E. M. Oksche A. Hein S. Yulis C. R. (1992). Cell biology of the subcommissural organ. *Int. Rev. Cytol.* 135 39–121. 10.1016/s0074-7696(08)62038-0 1618609

[B67] Rodríguez E. M. Rodríguez S. Hein S. (1998). The subcommissural organ. *Microsc. Res. Tech*. 41 98–123. 10.1002/(SICI)1097-0029(19980415)41:2<98::AID-JEMT2<3.0.CO;2-M 9579598

[B68] Rose C. D. Pompili D. Henke K. Van Gennip J. L. M. Meyer-Miner A. Rana R. (2020). SCO-Spondin defects and neuroinflammation are conserved mechanisms driving spinal deformity across genetic models of idiopathic scoliosis. *Curr. Biol.* 30 2363.e6–2373.e6. 10.1016/j.cub.2020.04.020 32386528

[B69] Sasai N. Kadoya M. Ong Lee Chen A. (2021). Neural induction: historical views and application to pluripotent stem cells. *Dev. Growth Differ.* 63 26–37. 10.1111/dgd.12703 33289091

[B70] Satoh N. (2008). An aboral-dorsalization hypothesis for chordate origin. *Genesis* 46 614–622. 10.1002/dvg.20416 18932262

[B71] Striedter G. F. Northcutt R. G. (2020). *Brains Through Time. A Natural History of Vertebrates.* London: Oxford University Press.

[B72] Troutwine B. R. Gontarz P. Konjikusic M. J. Minowa R. Monstad-Rios A. Sepich D. S. (2020). The reissner fiber is highly dynamic in vivo and controls morphogenesis of the spine. *Curr. Biol*. 30 2353.e3–2362.e3. 10.1016/j.cub.2020.04.015 32386529 PMC7891109

[B73] Vio K. Rodríguez S. Navarrete E. H. Pérez-Fígares J. M. Jiménez A. J. Rodríguez E. M. (2000). Hydrocephalus induced by immunological blockage of the subcommissural organ-Reissner’s fiber (RF) complex by maternal transfer of anti-RF antibodies. *Exp. Brain Res.* 135 41–52. 10.1007/s002210000474 11104126

[B74] Vio K. Rodríguez S. Yulis C. R. Oliver C. Rodríguez E. M. (2008). The subcommissural organ of the rat secretes Reissner’s fiber glycoproteins and CSF-soluble proteins reaching the internal and external CSF compartments. *Cerebrospinal Fluid Res*. 5:3. 10.1186/1743-8454-5-3 18218138 PMC2265671

[B75] Wagner C. Batiz L. F. Rodríguez S. Jiménez A. J. Páez P. Tomé M. (2003). Cellular mechanisms involved in the stenosis and obliteration of the cerebral aqueduct of hyh mutant mice developing congenital hydrocephalus. *J. Neuropathol. Exp. Neurol.* 62 1019–1040. 10.1093/jnen/62.10.1019 14575238

[B76] Wicht H. Lacalli T. H. (2005). The nervous system of amphioxus: structure, development and evolutionary significance. *Can. J. Zool.* 83 122–150. 10.1139/z04-163 33356898

[B77] Williams N. A. Holland P. W. H. (1996). Old head on young shoulders. *Nature* 383:490. 10.1038/383490a0

[B78] Yang S. Emelyanov A. You M. S. Sin M. Korzh V. (2021). Camel regulates development of the brain ventricular system. *Cell Tissue Res.* 383 835–852. 10.1007/s00441-020-03270-1 32902807 PMC7904751

